# Speciation Features of *Ferdinandcohnia quinoae* sp. nov to Adapt to the Plant Host

**DOI:** 10.1007/s00239-024-10164-1

**Published:** 2024-03-19

**Authors:** Zaki Saati-Santamaría, José David Flores-Félix, José M. Igual, Encarna Velázquez, Paula García-Fraile, Eustoquio Martínez-Molina

**Affiliations:** 1 Departamento de Microbiología y Genética, Universidad de Salamanca, Salamanca, Spain; 2 Present Address: Instituto de Investigación en Agrobiotecnología (CIALE), Universidad de Salamanca, Salamanca, Spain; 3 Institute of Microbiology of the Czech Academy of Sciences, Vídeňská, Prague, Czech Republic; 4 Instituto de Recursos Naturales y Agrobiología, IRNASA-CSIC, Salamanca, Spain; 5 Unidad Asociada Grupo de Interacción Planta-Microorganismo, Universidad de Salamanca-IRNASA-CSIC, Salamanca, Spain

**Keywords:** Speciation, *Ferdinandcohnia quinoae*, Quinoa, Adaptation, Comparative genomics, Microbial ecology

## Abstract

The bacterial strain SECRCQ15^T^ was isolated from seeds of *Chenopodium quinoa* in Spain. Phylogenetic, chemotaxonomic, and phenotypic analyses, as well as genome similarity indices, support the classification of the strain into a novel species of the genus *Ferdinandcohnia,* for which we propose the name *Ferdinandcohnia quinoae* sp. nov. To dig deep into the speciation features of the strain SECRCQ15^T^, we performed a comparative genomic analysis of the genome of this strain and those of the type strains of species from the genus *Ferdinandcohnia*. We found several genes related with plant growth-promoting mechanisms within the SECRCQ15^T^ genome. We also found that singletons of *F. quinoae* SECRCQ15^T^ are mainly related to the use of carbohydrates, which is a common trait of plant-associated bacteria. To further reveal speciation events in this strain, we revealed genes undergoing diversifying selection (e.g., genes encoding ribosomal proteins) and functions likely lost due to pseudogenization. Also, we found that this novel species contains 138 plant-associated gene-cluster functions that are unique within the genus *Ferdinandcohnia*. These features may explain both the ecological and taxonomical differentiation of this new taxon.

**Supplementary Information:**

The online version contains supplementary material available at 10.1007/s00239-024-10164-1.

## Introduction

Each earth ecosystem, including animals and plants, has their own distinctive microbial communities, which sometimes have evolved to develop important ecological functions (Nayfach et al. 2021; Debray et al. 2022; Hartmann and Six 2023). For instance, the plant microbiome is capable to benefit its host by providing access to nutrients and through the protection against biotic and abiotic factors (Ali et al. 2022; Chialva et al. 2022). Specially, endophytic microbes usually share a beneficial relation with their plant hosts (Flores-Félix et al. 2015; García-Fraile et al. 2015; Poveda et al. 2022). Indeed, some microbes have been adapted to the lifestyle in some specific plant tissues, and furthermore, some of them are vertically transmitted to descendants within seeds (Abdelfattah et al. 2022; Simonin et al. 2022). These seed symbionts may have been co-evolved to interact intimately with the host, and their study is of utmost importance to understand biological and ecological processes as well as to inspire advances in the development of biofertilizers (Dubey et al. 2021; Abdelfattah et al. 2022; Laranjeira et al. 2022; Simonin et al. 2022).

The evolution and adaptation into a new ecological niche are usually responsible of the bacterial speciation events (Baquero et al. 2021), and this evolution can be investigated through the analysis of the genomes and their gene content. For instance, it is known that many host-associated bacteria share common genes that facilitate competition, colonization, and evasion of host immune system (Wiesmann et al. 2022). In the case of the bacterial adaptation to the plant niche, there are few studies that provide insights into the processes (i.e., gain of genes, mutations) that lead the adaptation to this habitat (Levy et al. 2018a, b; Li et al. 2021a, b). However, it is not completely understood, not only how a bacterium evolves to gain fitness to the plant environment, but neither how a novel species arises in a differentiate niche.

Here we characterized an endophytic strain of quinoa seeds named SECRCQ15^T^ phylogenetically related to the *Ferdinandcohnia* species and whose genetic, chemotaxonomic, and phenotypic characteristics showed that it is a novel species of this genus which we propose to name as *Ferdinandcohnia quinoae* sp. nov. Through genome analyses, we characterize this species based on its putative roles in its plant host. Then, we show a comparative genomic study where we highlight the genomic features distinctive of the novel species *F. quinoae* with respect to the remaining species of genus *Ferdinandcohnia*, which were isolated from different ecosystems, such as air -*F. onubensis* (Dominguez-Moñino et al., 2018)-, compost -'*F. nitroreducens*' (Guo et al. 2016)-, human stools -'*F. sinesaloumensis*' and '*F. timonensis*' (Kokcha et al. 2012; Senghor et al. 2017)- and soil -*F. humi* (Heyrman et al. 2005), *F. salidurans* (Son et al. 2019) and *F. aciditolerans* (Ding et al. 2019)-. Our findings provide novel insights into the speciation features of a novel species occupying a different niche than the remaining species of the genus *Ferdinandcohnia*.

## Methods

### Strain Isolation

The strain SECRCQ15^T^ was isolated from *Chenopodium quinoa* seeds harvested from plants cultivated in Ciudad Rodrigo (Salamanca, Spain, 40° 35′ 02.6′′ N 6° 31′ 56.5′′ W). Quinoa seeds were surface sterilized with ethanol (70%) for 3 min and sodium hypochlorite (2%) for 2 min and were washed 5 times in sterile distilled water. An aliquot of the last wash water was plated on tryptic soy agar (TSA) and incubated at 28 °C for 48 h as a disinfection control, where no bacterial growth was observed. Surface sterilized seeds were crushed in a sterile mortar and resuspended in sterile water. Decimal dilutions from the suspension were obtained to isolate the endophytic bacteria and 100 μL of each suspension was spread on TSA plates (Sigma Co.) which were incubated at 28 °C for 48 h. Despite we have isolated other strains (data not shown), here we focus on the analysis of the strain SECRCQ15^T^ due to its taxonomic novelty. The strain was cryopreserved (− 80 °C; 25% glycerol solution) and plated when needed.

### Whole Genome Sequencing

We extracted DNA of the strain SECRCQ15^T^ after 2 days of growth in TSA (28 °C) using the Quick DNA Fungal/Bacterial Miniprep kit (Zymo Research, Irvine, CA, USA). The draft genome was sequenced on an Illumina NextSeq 500 Platform (75 pb Paired End). The genome contigs were assembled with SPAdes (Bankevich et al. 2012). The completeness and contamination levels were measured as previously detailed (Saati-Santamaría et al. 2022a) with BUSCO (Simão et al. 2015), and CheckM (Parks et al. 2015), respectively. We made both the structural and the functional genome annotation with RAST (v2.0) (Aziz et al. 2008).

### Phylogenetic Analyses

Amplification and sequencing of 16S rRNA gene were performed according to Carro et al. (2012) with some modifications. Briefly, 16S rDNA amplification was done with primers 27F (5′-AGAGTTTGATCTGGCTCAG-3′) (Weisburg et al. 1991) and 1522R (5′-AAGGAGGTGATCCANCC-3′) (Carro et al. 2012), using a REDTaq® ReadyMix™ (Sigma, USA) in 50 μl reaction volume following the manufacturer’s instructions. PCR products were purified directly from the gel with the GeneJET Gel Extraction and a DNA Cleanup Micro Kit (Thermo Scientific™, Göteborg, Sweden). Afterward, 16S rDNA PCR products were bidirectionally sequenced using a BigDye™ Terminator (v3.1) at the Sequencing DNA Service (NUCLEUS; University of Salamanca, Spain). Reads were aligned with SeqMan Pro software (DNASTART Inc., USA) to obtain the SECRCQ15^T^ 16S rDNA consensus sequence. This consensus sequence was compared to public sequences using BlastN (Altschul et al. 1990) (against the GenBank database) and EzTaxon-e (Kim et al. 2012) programs. The most closely related sequences from type strains were aligned with the Clustal_X software (Thompson et al. 1997) and distances were calculated according to Kimura´s two-parameter model (Kimura 1980) The phylogenetic trees were inferred using the neighbor joining (NJ) and maximum likelihood (ML) models (Saitou & Nei 1987; Rogers & Swofford, 1998). MEGA7 package (Kumar et al. 2016) was used for all analyses. We used all available gene and genomic sequences as available from the type strains of the *Ferdinandcohnia* species.

### Genome Analyses and Comparative Genomics

We measured the genomes relatedness through genome similarity indexes as detailed before (Saati-Santamaría et al. 2021). Briefly, we used PYANI software (v0.2.10) (Pritchard et al. 2016) to measure the ANIb values and the Genome-to-Genome Distance Calculator (GGDC v2.1) (Auch et al. 2010; Meier-Kolthof et al. 2013) to measure the digital DNA-DNA hybridization (dDDH). Average amino acid identity (AAI) was calculated with the online tool ANI/AAI-Matrix from Enveomics toolbox (Rodriguez-R and Konstantinidis 2016) with default settings, which uses MMSeqs2 for protein comparisons, a minimum query coverage of 50% and a minimum identity of 40% for AAI calculations. The phylogenomic tree was created with the UBCG (v3) tool (default settings) (Na et al. 2018) which created codon alignments (with MAFFT) and trees based on 92 housekeeping genes. These are universal bacterial core genes that have been proven to be valuable to infer phylogenomic relationship of bacteria (Na et al. 2018). Then, the tree was visualized and edited in the interactive tree of life (iTOL) tool (v5) (Letunic and Bork 2021).

We annotated the functions of the genes/proteins with KofamKOALA (Aramaki et al. 2020), PLaBAse (Patz et al. 2021) and antiSMASH (v6.0) (Blin et al. 2021). The comparative genomic analysis was done with Anvi’o (Eren et al. 2015). The Horizontal Gene Transfer (HGT) events were searched with HGTector2 (Zhu et al. 2014).

We used the pseudofinder.py command (Syberg-Olsen et al. 2022) to search pseudogenes in the SECRCQ15^T^. We used the whole Swissprot database as a reference and we retained the results with e value < 1e-4 (‘diamond’ search). As the input for this analysis, we used the GBK (gene bank format) annotation file provided by Prokka (Seemann 2014). This annotation was done with the ‘compliant’ and ‘rfam’ flags activated to enable the annotation of non-coding RNAs (ncRNAs) to eliminate false positive 'pseudogene' candidates. The genome the analysis of *dN*/*dS* was also done with pseudofinder, by using the ‘sleuth’ command. We used as a test the SECRCQ15^T^ genome, and the genomes of other *Ferdinandcohnia* species as a reference (run separately) (*Ferdinandcohnia aciditolerans* YN-1^T^, GCF_003640645.1; *Ferdinandcohnia humi* DSM 16318^T^, GCF_001439915.1; *Ferdinandcohnia onubensis* 0911MAR22V3^T^, GCF_002734215.1; ‘*Ferdinandcohnia timonensis’* MM10403188^T^, GCF_000285535.1). The results were classified as follows: positive selection: *dN/dS* > 1; neutral selection: *dN/dS* = 1; purifying selection: *dN/dS* < 1.

### Phenotypic and Chemotaxonomic Analyses

The strain was grown on nutrient agar (NA, Sigma Co.) for 48 h at 22 °C to check for motility by phase-contrast microscopy using the hanging drop method. Gram staining was carried out by the procedure described by Doetsch (1981) after 24 h of incubation at 28 °C. The flagellation type was determined by electron microscopy after 48 h of incubation of strain SECRCQ15^T^ on nutrient agar at 22 °C. The cells were gently suspended in sterile water and then stained with 2% uranyl acetate and examined at 80 kV with a Tecnai Spirit Twin transmission electron microscope.

The cellular fatty acids were analyzed by using the Microbial Identification System (MIDI; Microbial ID) Sherlock 6.1 and the library RTSBA6 according to the technical instructions provided by this system (Sasse 1990). The strains *F. humi* LMG 22167^ T^ and ‘*F. timonensis*’ DSM 25372^ T^ were included as reference, which were obtained from their corresponding culture collections: BCCM/LMG (https://bccm.belspo.be/about-us/bccm-lmg), and the German Collection of Microorganisms and Cell Cultures (DSMZ; https://www.dsmz.de/), respectively. The strains were grown on TSA plates (Becton Dikinson, BBL) for 48 h at 28 °C. Other chemotaxonomic analyses were carried out by the Identification Service of Leibniz Institute DSMZ (Deutsche Sammlung von Mikroorganismen und Zellkulturen GmbH, Braunschweig, Germany) for which the strain SECRCQ15^T^ was cultivated in TSB (Becton Dickinson, BBL) for 48 h at 28 °C and 180 rpm. The respiratory quinones and polar lipids were analyzed as described by Tindall (1990). To perform the analysis of peptidoglycan the whole cells of strain SECRCQ15^T^ were hydrolyzed with HCl at 100 °C during 15 h. The hydrolysates were subjected to thin-layer chromatography on cellulose plates using the solvent system of Rhuland et al. (1995).

The phenotypic characterization included the characteristics recommended in the minimal standards for aerobic endospore-forming bacteria (Logan et al. 2009) and was performed according to the methods described by Claus and Berkeley (1986) and by using API 20NE and API32GN systems (bioMerieux, France) according to the manufacturer’s instructions but adding MgSO_4_ to the media supplied by the kit, since this salt improves the growth of *Ferdinadcohnia* strains in these systems. The strain was unreactive in API 50CHB systems as occurred with its closest related taxon *F. humi* (Heyrman et al. 2005). The anaerobic growth was tested in fluid tetrathionate medium (Sigma, USA). Acetoin production, ability to grow in the presence of 2, 5, and 7% NaCl, nitrate reduction, phenylalanine deaminase, catalase, caseinase, gelatinase, amylase, and oxidase were analyzed as was described elsewhere (Claus and Berkeley 1986). Acid production from D-glucose, D-xylose, D-mannitol and L-arabinose and gas from glucose were analyzed in liquid medium as described previously (Claus and Berkeley 1986). Growth was determined at 4, 15, 25, 28, 37, 40, and 45 °C in TSA medium (Difco, BBL). The growth at pH 7 to 8 was tested in nutrient broth (Difco, BBL) containing 200 mM of Na_2_HPO_4_/NaH_2_PO_4_ and the growth at pH 9 and 10 was tested in the same medium containing 200 mM of NaHCO_3_/Na_2_CO_3_. The strain *F. humi* LMG 22167^T^ was included in the phenotypic study as reference.

## Results

### Genome Assembly

The assembly of the SECRCQ15^T^ genome yielded 265 contigs (L50 = 15; N50 = 89,801), and a total genome size of 4,443,130 bp, with 4351 coding region sequences (CDS) and 44 RNAs. The genomic G + C content is 36.0%. The genome contains 100% of complete BUSCO genes and 1.8 × contamination.

### Phylogenetic and Phylogenomic Location of the Strain SECRCQ15^T^

**Fig. 1:**
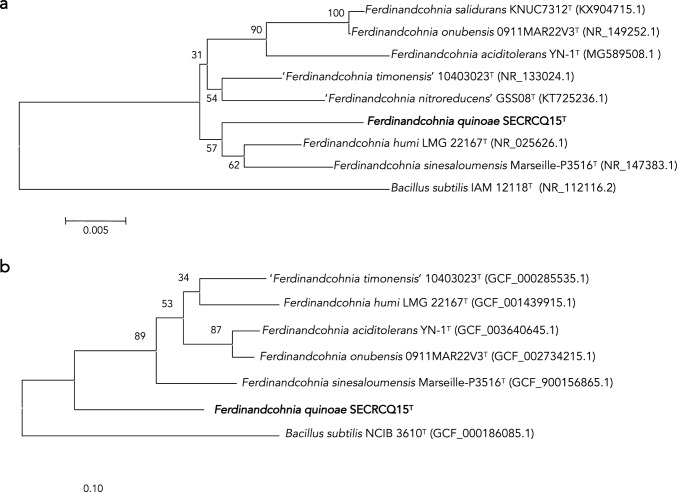
Phylogeny of the SECRCQ15^T^ strain. **a** Maximum likelihood phylogenetic tree based on nearly complete 16S rRNA gene sequence (1500 nucleotides) of *Ferdinandcohnia quinoae* SECRCQ15^T^ and the remaining species of the genus *Ferdinandcohnia*. *Bacillus subtilis* IAM 12118^T^ was used as outgroup. The significance of each branch is indicated by a bootstrap value calculated in percentage for 1000 subsets. Bar, 5 nt substitutions per 1000 nt. **b** Phylogenomic tree based on the consensus of 92 housekeeping gene phylogenies built with the UBCG pipeline. Bar, 100 nt substitutions per 1000 nt

First, we aimed to ensure the taxonomic placement of the strain SECRCQ15^T^. The comparison of the 16S rRNA gene sequence of this strain (1500 nucleotides) against those of type strains held in EzTaxon-e database indicated that it belongs to the genus *Ferdinandcohnia*, with *F. humi* DSM 16318^T^ and *“F. timonensis*” 10403023^T^ sharing the highest similarity, 98.1%, The remaining species shared similarity values lower than 98%; “*F. nitroreducens*” GSS08^T^ 97.8%, “*F. sinesaloumensis*,” *F. onubensis* and *F. salidurans* 97.4% and *F. aciditolerans* 96.9%. The phylogenetic analyses based on the 16S rRNA gene (both NJ and ML analyses), showed that the strain SECRCQ15^T^ formed a branch phylogenetically divergent from the remaining *Ferdinandcohnia* species (Fig. 1a). Similarly, the phylogenomic tree place the strain in a separate branch (Fig. 1b).

The genomes of the *Ferdinandcohnia* species showed less than 75% of ANIb values with respect to the genome of the strain SECRCQ15^T^ (Table 1) and the dDDH values were lower than 24% in all cases (Table 1). They are below threshold values used for bacterial species differentiation (Chun et al. 2018; Jain et al. 2018; Peral-Aranega et al. 2020; González-Dominici et al. 2021). The phylogenetic analyses and the phylogenomic tree based on 92 housekeeping genes confirmed that strain SECRCQ15^T^ represents a new species of genus *Ferdinandcohnia* for which we propose the name *Ferdinandcohnia quinoae* sp. nov. with SECRCQ15^T^ as type strain (Fig. 1). In addition, the chemotaxonomic and phenotypic features of this strain (shown in Supplementary material) support its classification as a different *Ferdinandcohnia* species.

**Table 1:** dDDH, ANIb, and AAI values (in percentage) shared among the studied type strains

	1	2	3	4	5	6
dDDH						
1	*					
2	19.7	*				
3	19.2	25.8	*			
4	18.8	26.4	46.3	*		
5	19.9	24.1	24	24	*	
6	19.3	26.7	25.9	26.5	23.8	*
ANIb						
1	*					
2	72.86	*				
3	72.63	80.9	*			
4	72.72	81.64	91.08	*		
5	73.01	79.69	80.02	80.45	*	
6	73.01	82.05	81.64	82.21	79.71	*
AAI						
1	*					
2	70.79	*				
3	70.27	83.18	*			
4	71.46	84.21	92.89	*		
5	70.97	82.39	83.36	84.11	*	
6	71.56	84.32	84.37	85.44	83.14	*

1. *Ferdinandcohnia quinoae* SECRCQ15^T^; 2. *Ferdinandcohnia humi* DSM 16318^T^ (GCF_001439915.1); 3. *Ferdinandcohnia aciditolerans* YN-1^T^ (GCF_003640645.1); 4. *Ferdinandcohnia onubensis* 0911MAR22V3^T^ (GCF_002734215.1); 5. '*Ferdinandcohnia sinesaloumensis*' Marseille-P3516^T^ (GCF_900156865.1); 6. ‘*Ferdinandcohnia timonensis*’ MM10403188^T^ (GCF_000285535.1)

### Novelty in the Gene Content Within the *Ferdinandcohnia pangenome*

**Fig. 2:**
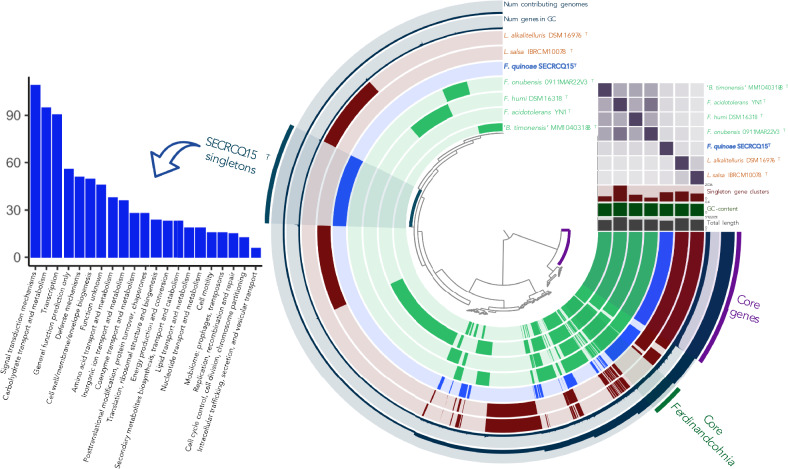
Comparative analyses of the *Ferdinandcohnia* genomes. The 7 inner circles show the presence/absence of Gene Clusters (GC) (homologous genes across genomes). Both the ANIb matrix and the GC presence/absence patterns support the differentiation of the *F. quinoae* species. We show below the functional categories of the SECRCQ15^T^ singletons, which serves as a summary of the metabolism that drove the ecological speciation of this new taxa

To shed light on the novel genomic/metabolic features of *F. quinoae* we performed a comparative genomic analysis of the strain SECRCQ15^T^ and the genomes of the closest related type strains. This analysis allowed to identify 1254 gene singletons in the SECRCQ15^T^ genome (genes unique to this strain) (Fig. 2). Out of these singletons, 862 were assigned to a COG (Cluster of Orthologous Groups of proteins) category, which are mainly related with signal transduction, carbohydrate metabolism, and transcription (Fig. 2).

We compared SECRCQ15^T^ gene clusters annotations (COGs) with those suggested to be related with plant-bacteria interactions in a previous comparative genomic study (Levy et al. 2018a, b). We used a list of *Bacillus* COGs that were significantly more abundant in plant isolates than in soil isolates. This search returned 138 of the SECRCQ15^T^ gene clusters (11.15%) as plant-associated COGs. In contrast, the core-genome of the *Ferdinancohnia* genus (Fig. 2) just contains 35 plant-associated COGs (8.64% of 405 core gene clusters). This enrichment of PA-functions of *F. quinoae* suggest an adaptative mechanism to live within the plant environment.

**Fig. 3:**
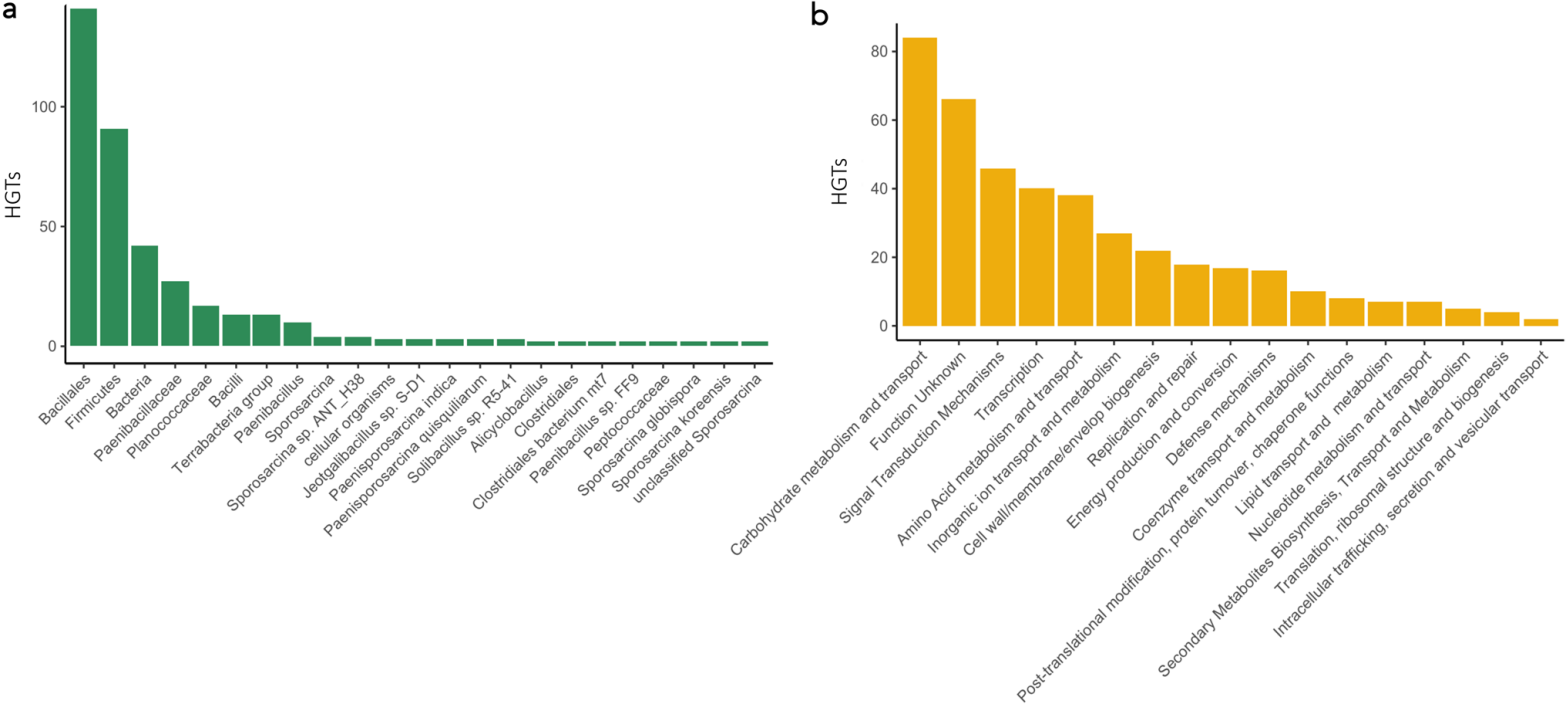
Putative horizontal gene transfer events found in the *Ferdinandcohnia quinoae* SECRCQ15^T^ genome. **a** Summary of the taxonomy of the donor strains for the transferred genes. **b** Summary of the metabolism categories of the horizontally acquired genes

Finally, we aimed to unveil what functions have been horizontally acquired by this strain and which microorganisms acted as donors. We found 422 HGT events within the SECRCQ15^T^ genome, which mainly come from closely related taxa (Fig. 3a), with a few exceptions, such as genes potentially acquired from the *Sporosarcina* genus (Caryophanales order), or from the *Peptococcaceae* family (Clostridia class). Much of these HGT are related with the metabolism of carbohydrates (84 genes) (Fig. 3b), or signal transduction (46 genes), but also many of them (66 genes) belong to COGs with no described function. Of these HGTs, 174 belonged to the singletons of the SECRCQ15^T^ strain, which represent 41% of the HGTs, and 13.8% of these singletons. This may indicate that most of the HGT events, since are unique for this strain, likely occurred recently and/or may be related with its speciation process, but still about 85% of the unique genes of the strain in the genus have an uncertain origin, such as gene diversification.

### Gene-Wide Evolution of *Ferdinandcohnia quinoae* sp. nov. SECRCQ15^T^

**Fig. 4:**
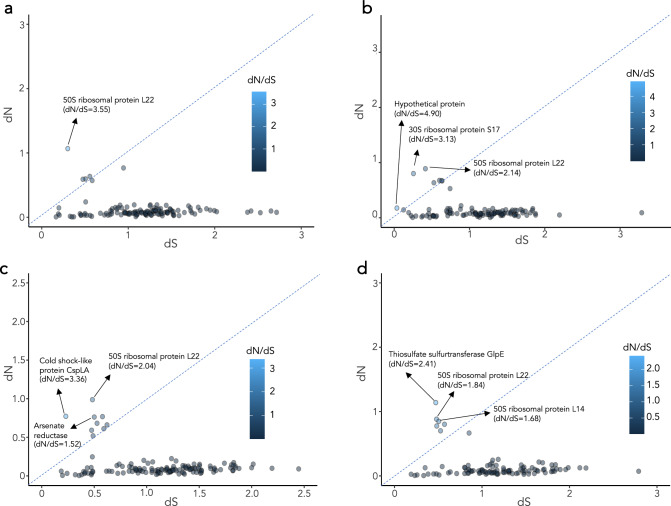
Gene-wide evolution analysis of the strain SECRCQ15^T^. *dN*/*dS* ratio of SECRCQ15^T^ genes using as a reference the genomes of other species in the genus: **a** *Ferdinandcohnia aciditolerans* YN-1^T^ (GCF_003640645.1); **b** *Ferdinandcohnia humi* DSM 16318^T^ (GCF_001439915.1); **c** *Ferdinandcohnia onubensis* 0911MAR22V3^T^ (GCF_002734215.1); **d** ‘*Ferdinandcohnia timonensis’* MM10403188^T^ (GCF_000285535.1)

We explored the degree of selection acting on protein-coding genes by means of the *dN*/*dS* ratio, which represents the non-synonymous (*dN*) to synonymous (*dS*) nucleotide substitution rate. We found 4–9 genes (depending on the reference species used for this analysis) that are under positive (diversifying) selection (*dN*/*dS* > 1), of which 1–3 show values > 2 (Fig. 4). Interestingly, we found a high positive selection acting on genes encoding ribosomal proteins, which help to structure the ribosomal RNA. We also found other functions being diversified, such as the Cold shock-like protein CspLA, a thiosulfate sulfotransferase (GlpE), and an arsenate reductase (*arsC*). The evolution of these genes might be related with some selective pressure related with the SECRCQ15^T^ plant niche.

Beyond diversification, we also investigated to what extend some functions have been lost in the SECRCQ15^T^ genome due to pseudogenization, which might reveal functions that are not needed within this strain and that can help to understand their speciation events. We found 219 pseudogenes (5% of the total open reading frames) (Table 2). Most of these lost functions were unknown, related with signal transduction mechanism, transcription or to cell wall biogenesis, among others (Fig. S3).

**Table 2:** Pseudogenes found in the SECRCQ15^T^ strain

	Count of genes
Pseudogenes (total):	219
Pseudogenes (too short):	131
Pseudogenes (too long):	81
Pseudogenes (fragmented):	5
Pseudogenes (no predicted ORF):	2
Pseudogenes (frameshift):	0
Pseudogenes (missing start codon):	0
Pseudogenes (missing stop codon):	0
Pseudogenes (internal stop codon):	0
Pseudogenes (multiple issues):	0
Intact genes:	4153

### Ecological Features of the SECRCQ15^T^ Strain

To unveil potential ecological functions of the new proposed species, we analyzed its genome to find genes likely related with the interaction with its host plant and/or the surrounding microbiome. We found enzymes related with plant hormone biosynthesis, such as an Indole-3-acetamide amidohydrolase and an Indole-3-acetaldehyde:NAD + oxidoreductase, both proteins involved in indolacetate (IAA) synthesis. There are also proteins encoded that are related with the production of dimethylallyl diphosphate and geranyl-PP from D-Glyceraldehyde 3-phosphate (glycolysis product). These two metabolites are substrates for the biosynthesis of zeatin and monoterpenes, respectively, and could be incorporated into the plant biosynthetic pathways. Also, this strain could boost plant systemic response due to the presence of the microbial associated molecular patterns (MAMPs) flagellin (Flg22) and Elongation Factor-TU (Ef-TU). The presence of genes encoding an acetolactate synthase, responsible for the synthesis of acetoin represents, not only a putative role in ISR elicitation, but also another plant growth-promoting mechanism. Additionally, the genome encodes alkaline phosphatases (EC 3.1.3.1) and diverse pyrophosphatases (EC 3.6.1.1), which, together with the action of acids likely produced by this strain (i.e., there are genome evidences of malic acid biosynthesis) can help to release phosphorous accessible for the plant absorption. We also found complete pathways involved in vitamin biosynthesis, such as thiamine (vit B1), riboflavin (vit B2), nicotinate and nicotinamide (vit B3), pantothenate (vit B5), folate (vit B9), and menaquinone (vit K2). Finally, this strain may have a role in the sulfur cycle, since it encodes the proteins Sat, CysC, CysH, and CysII, responsible of the complete assimilatory sulfate reduction to sulfide. The genome does not show any putative role of this strain in atmospheric carbon (CO_2_) or methane (CH_4_) fixation.

We found that that strain SECRCQ15^T^ has a total of 2287 putative genes associated with bacterial plant growth-promoting traits “PGPTs,” according to the PLaBAse database. Of these, 70.4% are coincident with indirect mechanisms such as colonization, competition in the rhizosphere or resistance to stresses. While 29.6% corresponds to direct plant promotion mechanisms such as the genes already detected associated with auxin production, vitamin biosynthesis or the production of organic acids from different sugars. We also detected the *miaA* and *miaB* genes, isopentenyltransferase and methylpentenyltransferase, respectively, that are essential for the biosynthesis of cytokinins. Also, the genes *gabD* and *gabT*, which catabolise the degradation of gamma-aminobutyrate (GABA) on succinate were annotated on its genome. In addition, this analysis revealed an extensive genetic background for resistance and adaptation to the environment. This adaptation skill is supported by 204 genes involved in the degradation of xenobiotics such as the *paa* cluster for phenylacetate degradation, or the degradation of dichloropropene (adhP-propanol dehydrogenase); and resistance to metals such as arsenic and antimony (*arsA*, *arsB*, *arsC*, *arsR*), copper (*cusR*), zinc (*znuABC*) or lead (*prbB*), among others.

We searched for biosynthetic gene clusters (BGCs) that may produce any known specialized metabolite, but the search just allowed to detect already undescribed BGCs with no similarity against those available in the MiBIG and antiSMASH databases: a lasso peptide, a type III PKS, a hybrid linear azol(in)e-containing peptide (LAP) and a ribosomally synthesized and post-translationally modified peptide product (RiPP). Due to its novelty, these BGCs may produce novel secondary metabolites with ecological or biotechnological interest.

## Discussion

In this work we have characterized one endophytic strain isolated from quinoa seeds obtained from a local farmer (Salamanca, Spain). The accurate assignment of this strain to a taxon was addressed by diverse phylogenetic and phylogenomic analyses and by physiological, phenotypic and chemotaxonomic approaches. The 16S rRNA gene analysis continues to be essential to place the bacterial isolates into a genus and to know their closest related species, which in the case of strain SECRCQ15^T^ were those of genus *Ferdinandcohnia*. The species of this genus were initially included within the genus *Bacillus*, which was subject to a deep reclassification recently (Gupta et al. 2020), being the new species described in this work, *F. quinoae* sp. nov., the first *Ferdinandcohnia* species described after this reclassification, which was based on phylogenomic analyses.

To deeper analyze both the taxonomic assignment of strain SECRCQ15^T^ and its potential role as a quinoa seed endophyte, we obtained its genome sequence, which is the first available genome of a quinoa endophytic strain. The analysis of this genome confirmed that this strain belongs to a new species of genus *Ferdinandcohnia,* being its closest phylogenetically related type strains those of *F. humi* DSM 16318^T^, which was isolated from an agricultural soil (Heyrman et al. 2005), and “*F. timonensis*” 10403023^T^, which was isolated from human stools (Kokcha et al. 2012). Several phenotypic and chemotaxonomic differences between these strains support that they are different species, and thus, we propose the description of *Ferdinandcohnia quinoae* sp. nov.

Considering that the proposed novel species expand the host-range of the genus *Ferdinandcohnia* to include plant seeds, we discussed its genome innovations and features that may fulfill the evolutionary process to fulfill its adaptation to that environment and its ecological roles in there. We found a large proportion of singletons within the *F. quinoae* SECRCQ15^T^ sp. nov. genome that are related with the carbohydrate metabolism. This functional expansion can be related with the broad availability of sugars and complex carbohydrates found within the seed and plant environment (Sasse et al. 2018; McLaughlin et al. 2023). Similarly, this set of genes encompass many plant-associated genes (Levy et al. 2018a, b), which also support its drift toward the plant lifestyle. Also, the enrichment of singletons related with transcription and signal transduction mechanisms may reflect a deeper speciation, not only in coding-sequences, but also in the regulation of cell functions, which outcomes could be investigated trough transcriptomics or other -omic data.

We also found several functions undergoing positive selection, such as ribosomal proteins. These mutations may have been driven by the need to interact with other biomolecules, and may affect ribosome assembly, leading to extensive alterations in both transcriptomic and proteomic profiles (Gómez et al. 2017). Diversifying selection on arsenate reductase and thiosulfate sulfurtransferase might be related with an improved metabolism of arsenate and thiosulfate. In the context of plant-bacteria interactions, some bacteria activate arsenic detoxification mechanisms. This can be relevant in environments where plants are exposed to arsenic contamination, as bacteria may contribute to the transformation of arsenate into a less toxic form, potentially influencing the overall arsenic bioavailability to plants (Cavalca et al. 2010). Thiosulfate can serve as a sulfur source for both plants and bacteria. Some bacteria may produce thiosulfate as a byproduct. In turn, plants can take up thiosulfate as a sulfur nutrient. Hence, thiosulfate sulfurtransferase diversification might lead to a enhanced sulfur metabolism, which might influence plant health and growth (Nakajima et al. 2019; Ranadev et al. 2023).

Beyond their speciation events, the complete genome of *F. quinoae* SECRCQ15^T^ revealed ecological features related with host-microbe interactions, concretely with beneficial plant-bacteria interactions. Further experiments will serve to elucidate its functions within quinoa seeds or developed plants. Similarly, the novelty of the BGCs related with the production of specialized metabolites, not only uncover ecological features (Saati-Santamaría, 2023), but also suggests this strain as a promising one for natural product research (Kalkreuter et al. 2020; Hemmerling & Piel 2022; Saati-Santamaría et al. 2022b).

## Conclusion

In sum, we provide an indepth-analyses of the speciation features of the strain SECRCQ15^T^. Both the phylogenies and the functional differentiation of this strain support its classification as a novel species. The ecological functions encoded within the *F. quinoae* sp. nov. genome will help to further understand the bacterial communities of quinoa plants. Also, this work expands the host range of the *Ferdinandcohnia* genus. The genomic analyses revealed signs of functional adaptation to the plant environment in the *F. quinoae* type strain and explain its adaptation within this niche.

## Protologue

Description of *Ferdinandcohnia quinoae* gen. nov. sp. nov.

*Ferdinandcohnia quinoae* (qui.no'ae. N.L. gen. n. quinoae, of quinoa).

Cells of the strain SECRCQ15^T^ were straight, aerobic, motile Gram-stain positive rods (width 0.6–0.8 µm, length 3.0–5.0 µm). Oval subterminal endospores were formed in swollen sporangia. Catalase and oxidase positive. Colonies of this strain on nutrient agar medium are opaque, white cream colored, raised with entire margins and smooth surfaces. Anaerobic growth was negative. It grows from pH 6 to pH 9 (optimal pH is 7). It can grow in the presence of 4% NaCl. It grows from 12 to 44 °C (optimal temperature is 30 °C). Nitrate is reduced to nitrite. Production of β-galactosidase and hydrolysis of aesculin are positive. Production of indole, urease, arginine dehydrolase, and H_2_S is negative. Production of gelatinase is weak. In API 20NE Assimilation of glucose, maltose, mannose, and gluconate is positive and that of L-arabinose, mannitol, N-acetyl-glucosamine, caprate, adipate, malate, citrate, and phenylacetate is negative. In API ID32GN glucose, melibiose, D,L-lactate, glycogen, maltose, and 3-hydroxi-butyrate are assimilated, but the assimilation of L-rhamnose, N-acetyl-glucosamine, D-ribose, inositol, sucrose, mannitol, salicin, L-fucose, D-sorbitol, L-arabinose, itaconate, suberate, propionate, caprate, valerate, citrate, malonate, acetate, 2 and 5 keto-gluconate, 3 and 4 hydroxi-benzoate, L-serine, L-alanine, L-histidine, and L-proline is negative. The major quinone is MK-7. Mesodiaminopimelic acid was not detected in the peptidoglycan. The lipid profile consists of diphosphatidylglycerol, phosphatidylglycerol, phosphatidylethanolamine, one unidentified aminophospholipid, and one unidentified phospholipid. The major fatty acids are iso-C_15:0_ and anteiso-C_15:0_. The G + C content of the strain SECRCQ15^T^ is 35.96 mol%. The type strain SECRCQ15^T^ (= LMG 32511^ T^ = CECT 30513^T^) was isolated from seeds of quinoa (*Chenopodium quinoa*) in Spain. The 16S rRNA gene and genome sequence were deposited at DDBJ/EMBL/GenBank under accession numbers OM791795 and JAKTTI000000000, respectively.

### Supplementary Information

Below is the link to the electronic supplementary material.

Supplementary file1 (PDF 2461 kb)

Supplementary file2 (PDF 248 kb)

## Data Availability

The genome sequence is available under the NCBI (https://www.ncbi.nlm.nih.gov/) bioproject PRJNA807880 (BioSample: SAMN26011952; Assembly: GCA_022427965.1; Genome nucleotide sequences: JAKTTI000000000). The 16S rRNA gene sequence is available through the following accession number: OM791795.
