# Supplementary material for: Speciation Features of Ferdinandcohnia quinoae sp. nov to Adapt to the Plant Host
Source: J Mol Evol. 2024 Mar 19;92(2):169–80. doi: 10.1007/s00239-024-10164-1 (PMC10978704; doi:10.1007/s00239-024-10164-1)
Supplement: Supplementary file 1 — Supplementary file1 (PDF 2461 kb) [file 239_2024_10164_MOESM1_ESM.pdf]

Supplementary Material

Speciation features of *Ferdinandcohnia quinoae* sp. nov to the plant host

Zaki Saati-Santamaría[1,2,3], *José David Flores-Félix*[1], *José M. Igual*[4,5], *Encarna Velázquez*[1,2,5],

Paula García-Fraile[1,2,*], *Eustoquio Martínez-Molina*[1,2,5]

[1]Departamento de Microbiología y Genética, Universidad de Salamanca, Salamanca, Spain.
[2]Instituto de Investigación en Agrobiotecnología (CIALE), Universidad de Salamanca, Salamanca, Spain.
[3]Institute of Microbiology of the Czech Academy of Sciences, Vídeňská, Prague, Czech Republic
[4]Instituto de Recursos Naturales y Agrobiología, IRNASA-CSIC, Salamanca, Spain.
[5]Unidad Asociada Grupo de Interacción Planta-Microorganismo, Universidad de Salamanca-IRNASA-CSIC, Salamanca, Spain.

Corresponding author: Paula García-Fraile, paulagarciafraile@usal.es

Content: This file contains supplementary results related with the chemotaxonomic and phenotypic features of the strain SECRCQ15^T

Chemotaxonomic and phenotypic description of the strain SECRCQ15^T

The chemotaxonomic analyses showed that menaquinone 7 (MK7) was the major respiratory quinone (99.4%) detected in strain SECRCQ15^T. It displayed a lipid profile (Supplementary Figure S1) consisting of diphosphatidylglycerol (DPG), phosphatidylglycerol (PG), phosphatidylethanolamine (PE), one unidentified aminophospholipid (APL) and one unidentified phospholipid (PL). Mesodiaminopimelic acid (DAP) was not detected in the peptidoglycan of strain SECRCQ15^T. The major fatty acids were iso-$C_{15:0}$ (36.9%) and anteiso-$C_{15:0}$ (36.8%). The comparison of the fatty acid profile of strain SECRCQ15^T mainly differed with respect to that of its closest relatives in the proportions of iso-$C_{14:0}$, iso-$C_{17:0}$ and $C_{16:1}\omega 11c$ (Table S1).

The results of the phenotypic characterization are given in the species description (protologue). Overall, these phenotypic features are distinctive from those of the closest type strains (Table S2). The strain SECRCQ15^T formed colonies white cream, round, smooth and convex with approximate diameters of 0.2-1 mm in TSA. It was Gram-stain positive, motile by means of peritrichous flagella and formed oval subterminal endospores swollen the sporangium (Supplementary Figure S2).

Table 2. Cellular fatty acid composition of strain SECRCQ15^T and its closest related type strains. Strains: 1. *Ferdinandcohnia quinoae* SECRCQ15^T; 2. *Ferdinandcohnia humi* LMG 22167^T; 3. '*Ferdinandcohnia timonensis*' DSM 25372^T Fatty acids present in both species in amounts lower than 1% are not shown. nd. not detected. Data are from this study.

Fatty acid	1	2	3
**Saturated straight-chain**			
$C_{14:0}$	1.2	1.1	4.3
$C_{16:0}$	0.8	0.8	2.0
**Saturated iso-branched**			
$C_{14:0}$	3.8	10.3	8.0
$C_{15:0}$	36.9	31.7	28.4
$C_{16:0}$	3.9	1.6	3.8
$C_{17:0}$	2.2	0.4	0.9
**Saturated anteiso-branched**			
$C_{15:0}$	36.8	40.1	37.9
$C_{17:0}$	4.9	1.7	3.7
**Unsaturated**			
$C_{16:1}\omega 11c$	1.8	3.5	5.5
$C_{16:1}\omega 7c$ alcohol	3.9	4.8	3.3
$C_{17:1}$ iso$\omega 10c$	1.8	0.7	0.6
Summed feature 4‡	1.6	1.4	0.9

‡Summed feature 4: $C_{17:1}$ anteiso B/ $C_{17:1}$ iso I

Table 3. Differential phenotypic characteristics of strain SECRCQ15^T and its closest related type strains Strains: 1, *Ferdinandcohnia quinoae* SECRCQ15^T; 2, *Ferdinandcohnia humi* DSM 16318^T; 3. '*Ferdinandcohnia timonensis*' DSM 25372^T. Data are from this study. +: positive, -: negative, w: weakly positive.

Characteristics	1	2	3
L-arabinose	-	+	+
N-acetyl-glucosamine	-	+	+
Melibiose	+	-	+
Gluconate	+	-	-
Glycogen	+	-	+
Malate	-	+	w
Citrate	-	+	+
Ribose	-	+	+
Sucrose	-	+	+
Salicin	-	+	+
L-fucose	-	+	-
Valerate	-	+	w
2-Keto-gluconate	-	+	w
5-Keto-gluconate	-	+	-

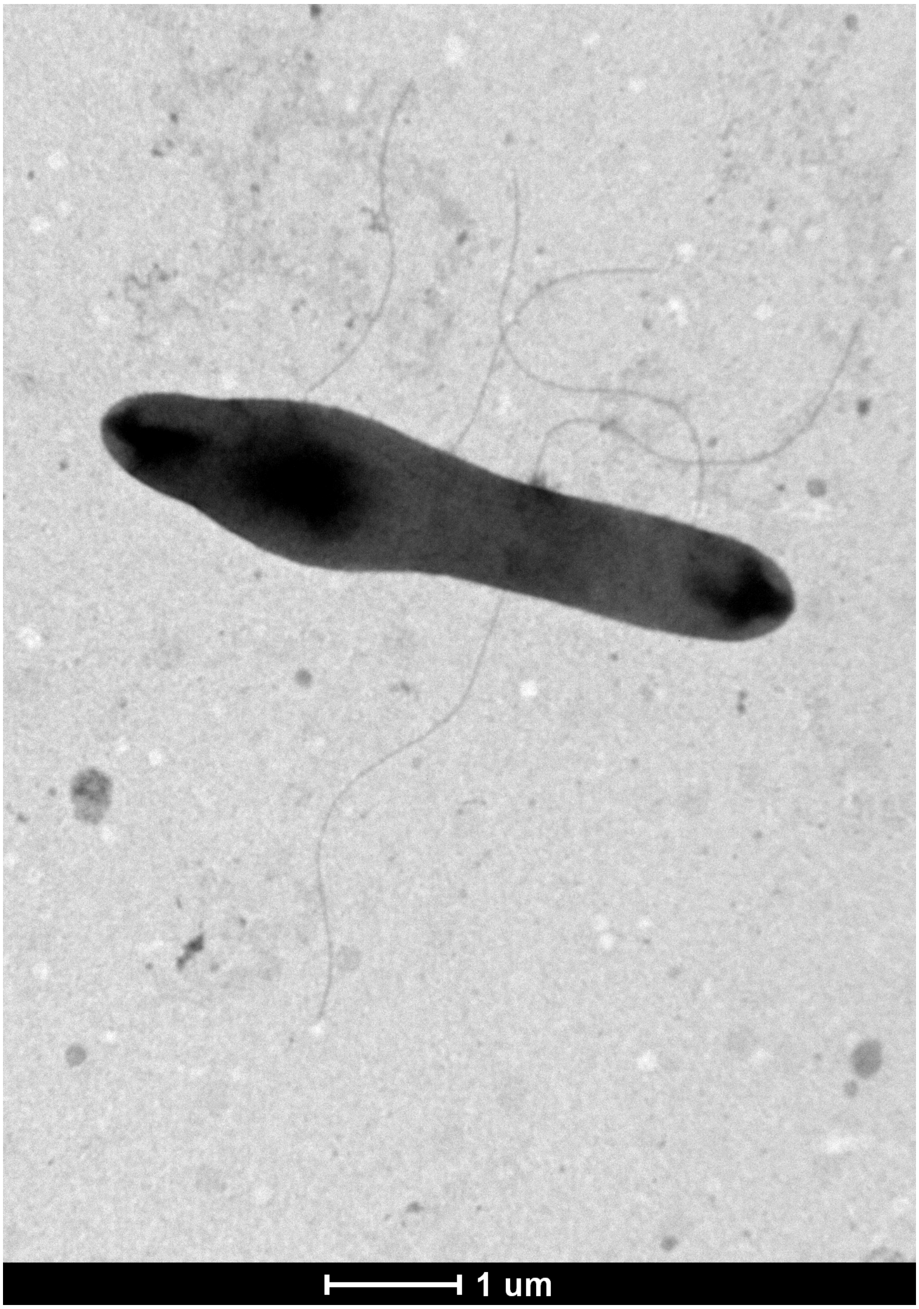

Figure S1. Electron micrograph of *Fredinandcohnia quinoae* SECRCQ15^{T} showing the peritrichous flagella (TEM) and the oval subterminal endospores swollen the sporangium.

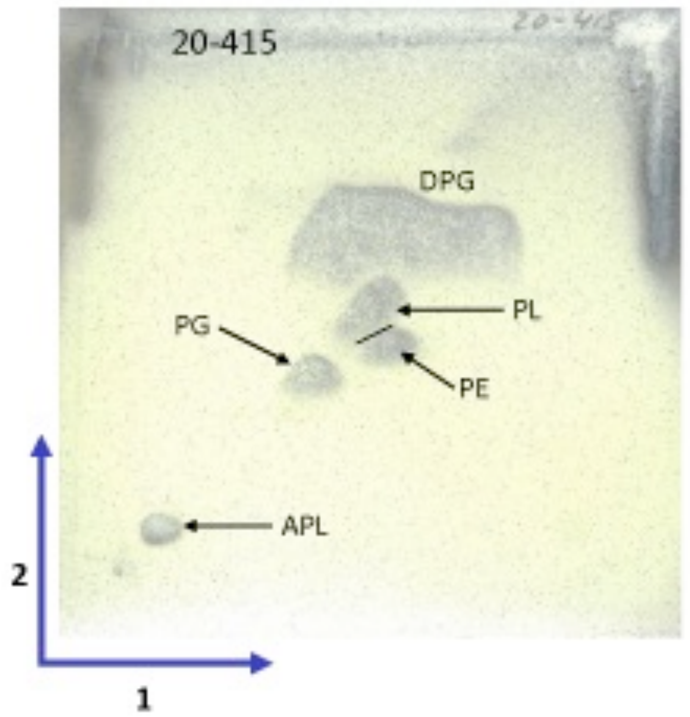

Figure S2. Polar lipids profile of strain *Fredinandcohnia quinoae* SECRCQ15^{T}. DPG: diphosphatidylglycerol; PG: phosphatidylglycerol; PE: phosphatidylethanolamine; APL: unidentified aminophospholipid; PL: unidentified phospholipid.
