# Supplementary figures and images for: Speciation Features of Ferdinandcohnia quinoae sp. nov to Adapt to the Plant Host
Source: J Mol Evol. 2024 Mar 19;92(2):169–80. doi: 10.1007/s00239-024-10164-1 (PMC10978704; doi:10.1007/s00239-024-10164-1)

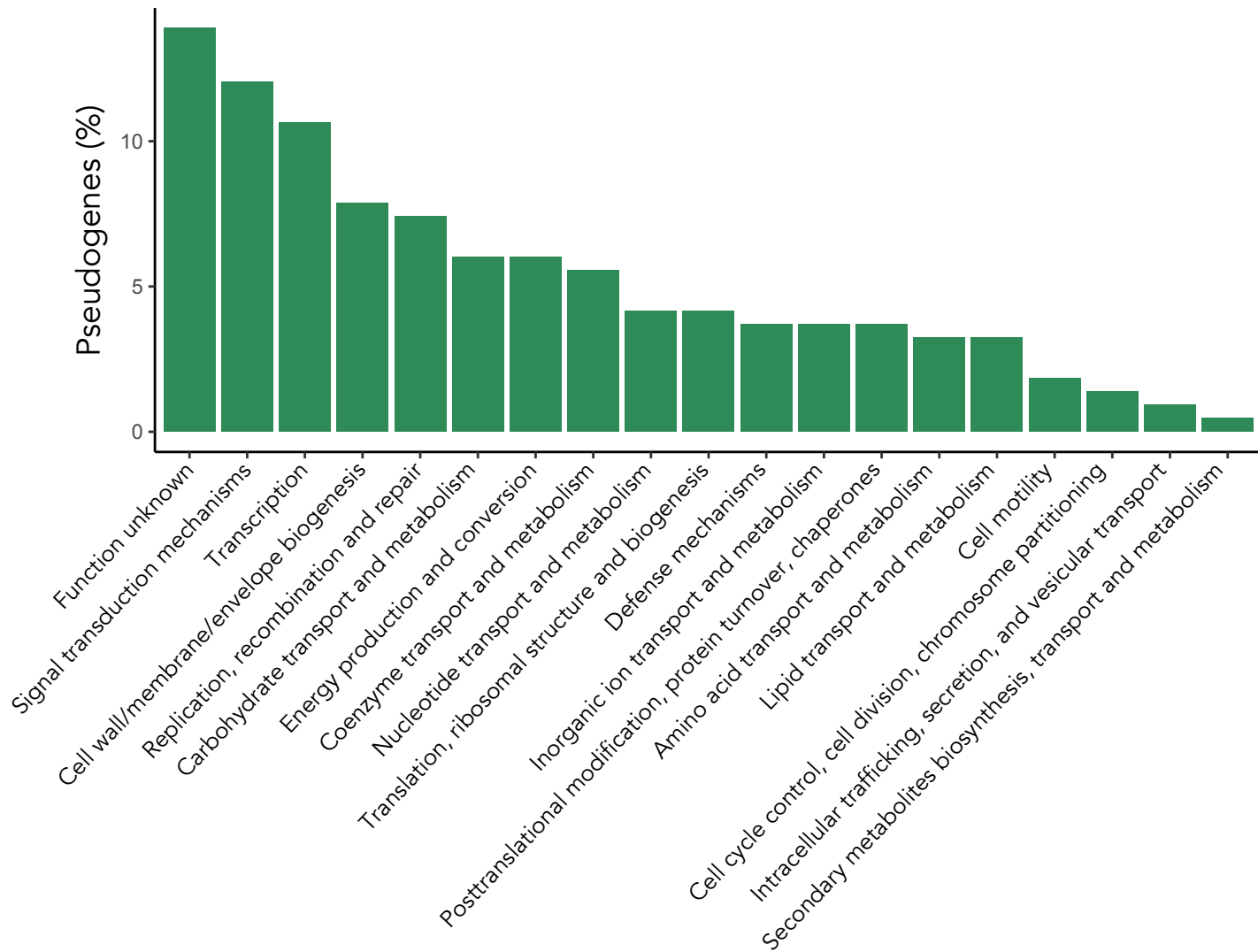

Supplement: Supplementary file 2 — Supplementary file2 (PDF 248 kb) [file 239_2024_10164_MOESM2_ESM.pdf]
